# Addressing the Path-Length-Dependency Confound in White Matter Tract Segmentation

**DOI:** 10.1371/journal.pone.0096247

**Published:** 2014-05-05

**Authors:** Matthew G. Liptrot, Karam Sidaros, Tim B. Dyrby

**Affiliations:** 1 Danish Research Centre for Magnetic Resonance, Centre for Functional and Diagnostic Imaging and Research, Copenhagen University Hospital Hvidovre, Copenhagen, Denmark; 2 Department of Computer Science, University of Copenhagen, Copenhagen, Denmark; University of Minnesota, United States of America

## Abstract

We derive the Iterative Confidence Enhancement of Tractography (ICE-T) framework to address the problem of path-length dependency (PLD), the streamline dispersivity confound inherent to probabilistic tractography methods. We show that PLD can arise as a non-linear effect, compounded by tissue complexity, and therefore cannot be handled using linear correction methods. ICE-T is an easy-to-implement framework that acts as a wrapper around most probabilistic streamline tractography methods, iteratively growing the tractography seed regions. Tract networks segmented with ICE-T can subsequently be delineated with a global threshold, even from a single-voxel seed. We investigated ICE-T performance using ex vivo pig-brain datasets where true positives were known via in vivo tracers, and applied the derived ICE-T parameters to a human in vivo dataset. We examined the parameter space of ICE-T: the number of streamlines emitted per voxel, and a threshold applied at each iteration. As few as 20 streamlines per seed-voxel, and a robust range of ICE-T thresholds, were shown to sufficiently segment the desired tract network. Outside this range, the tract network either approximated the complete white-matter compartment (too low threshold) or failed to propagate through complex regions (too high threshold). The parameters were shown to be generalizable across seed regions. With ICE-T, the degree of both near-seed flare due to false positives, and of distal false negatives, are decreased when compared with thresholded probabilistic tractography without ICE-T. Since ICE-T only addresses PLD, the degree of remaining false-positives and false-negatives will consequently be mainly attributable to the particular tractography method employed. Given the benefits offered by ICE-T, we would suggest that future studies consider this or a similar approach when using tractography to provide tract segmentations for tract based analysis, or for brain network analysis.

## Introduction

Diffusion weighted imaging (DWI) provides a novel and unique method with which to study white-matter microstructure within the brain (for an overview see [1] and [2]). In particular, processing of DWI data can produce estimates of white matter fibre directions ([3], [4], [5], [6]) from which voxelwise uncertainty orientation distribution functions (uODFs) can be generated. Probabilistic streamlining methods thereafter permit generation of connection-confidence maps e.g. Probabilistic Index of Connectivity (PICo) maps [9], by counting the relative propagating success of streamlines from a given seed region ([7], [8] and [9]). Such PICo maps can then be used to inform brain connectivity ([10], [11], [12]).

However, several well-documented confounds and limitations make it challenging to infer from such connection-confidence maps. These include confounds such as Path-Length Dependency (PLD) and the Partial Volume Effect (PVE), modelling limitations in regions of crossing fibres, and data limitations due to an image resolution that is far coarser than the axons themselves. These issues hamper the inference of any robust form of true anatomical connectivity (in terms of parallel associated axons) from these connection-confidence maps ([13], [14]).

Furthermore, the application of tract-specific statistics, and therefore also many forms of network analysis (e.g. [15]), relies upon the correct segmentation of tracts from PICo-type maps, usually by the application of a priori information in the form of waypoints, followed by a global threshold. Due to the reasons outlined above, this often proves to be problematic, and a single global threshold is generally insufficient. The PLD confound is one of the key obstacles to the application of global thresholding, and its inherent presence in all probabilistic streamline tractography methods ([9], [13]) imposes a great challenge to the segmentation of any tract system from a relevant seed region.

The origin of the PLD effect lies in the mechanics of the probabilistic streamline tractography method, and is simply due to the step-wise dispersion of the propagating streamlines along the length of a tract. Hence image resolution is also a contributory factor. PLD is an inherent side-effect of the probabilistic approach and is manifested as a monotonic, non-linear down-modulation of the calculated probability as a function of the propagation distance from the seed point ([9], [13]). Hence the resultant probability values produced per voxel represent only the chance that an average streamline could propagate thereto from the seed region, and cannot represent any anatomical connection strength of the tract – see [13] for a discussion. The consequential decline in the number of streamlines that manage to successfully propagate to distal portions of a tract means that those that do are too few to sufficiently sample uODFs therein.

The PLD effect is a well-known phenomenon and has been previously reported in early tractographic human studies, [9]. The problems related to PLD have since been demonstrated in a validation study [16], where they observed long WM tracts terminating arbitrarily depending upon the threshold chosen. The consequences of PLD can therefore be significant modulation of tractographic results, thereby also impacting upon subsequent diagnostic interpretation. Nevertheless, PLD has so far received little attention.

Whilst few studies have looked into moderating PLD itself, some attempts have been made to compensate for its effects. Normalisation by a distance-correction factor has been suggested ([17], [18]), but due to tract specific differences (different routes, different number of dispersions and anatomical complexities encountered) this cannot guarantee the removal of all PLD effects.

To be able to delineate specific longer tracts, many studies use constrained tractography (using a priori information such as waypoints) to enable a global threshold. Although this does not solve the PLD issue, the extra a priori info can help to delineate specific tracts. This approach was used together with additional heuristics by Sherbondy et al. [19] to counter the resistance to conventional thresholding of specific tracts due to the effect of PLD. It must be noted that such heuristics, together with the more general and commonly-used approach of applying waypoints, exclusion masks and termination regions, do not provide PICo map outputs, nor attempt to remove PLD itself, but can be useful to delineate specific tracts of interest.

Here, we visualise the PLD effect and its non-linear behaviour. We propose a heuristic method, based upon probabilistic tractography, to segment out a given tract system emanating from a seed region, with minimal influence from PLD. Based upon a region-growing approach, Iterative Confidence Enhancement for Tractography (ICE-T) is an easy-to-implement framework that is applicable as a wrapper function to most probabilistic streamline tractography methods. This work introduces ICE-T, its modifiable parameters (termed *ICE-T_threshold_* and *ICE-T_streams_*) and their generalizability, and investigates the method's parametric behaviour. We confirm both the applicability of our ICE-T method against the results derived by [16], via the use of the same ex vivo pig brain dataset that uniquely combined tractography with invasive tracer studies, and also its reproducibility. Additionally we demonstrate that, due to the non-linear nature of PLD, linear compensation methods may not be sufficient. Finally, we demonstrate the application of the ICE-T method to a human in vivo dataset.

## Theory

We define PLD as being the drop in connection confidence along a tract due to a combination of effects caused by the stepwise dispersion of streamlines, which is in turn due to the stepwise sampling of the uODFs and the compounding effect of anatomical complexities. ICE-T aims to significantly reduce PLD in existing probabilistic streamline tractography methods. The underlying principle of ICE-T is to ensure that the uODF of each voxel that is determined as being connected to the seed is sufficiently sampled by a suitably large number of streamlines. To achieve this we apply ICE-T to conventional tractography in order to grow the given seed region iteratively along its connections. This can be viewed as a tract segmentation step via region-growing, using a predicate of voxelwise connectivity determined by probabilistic tractography. The resultant grown seed region thereby represents the segmented tract system emanating from the given seed. Hereafter one can either use the region for tract-based analysis, or use it as a seed region for tractography. The outcome of the latter will not be a PICo map, but instead a map describing how well connected each voxel of the segmented tract is to all the other voxels within the tract. This can therefore be considered as a tract-based ACM [20], to which a global threshold can then be directly applied without the need to compensate for bias introduced by PLD.

Hereunder, we describe the mechanics of the ICE-T framework.

### ICE-T Framework

A tractography pipeline generally includes the following steps: voxelwise fibre-reconstruction and generation of uODFs, followed by the streamlining tracking process. ICE-T is a simple modification of this, introducing a feedback loop around the streamlining tracking process, and is described as pseudo-code in Table 1. ICE-T utilises the same parameters as tractography, with two exceptions: the number of streamlines generated per voxel is modified (ICE-T_streams_), and the threshold applied to the end-of-iteration connection-confidence map (ICE-T_threshold_).

**Table 1:** The ICE-T Framework as Pseudo-Code.

Stage	Task	Stage Inputs	Stage Outputs
1	Start	Iteration, i = 1	
		ICE-T_ROI_i_ = original seed region	
		ICE-T_streams_ parameter (e.g. 20)	
		ICE-T_threshold_ parameter (e.g. 0.01)	
		Global Threshold Parameter (e.g. 0.005)	
2	Perform streamlining from ICE-T_ROI_i_	ICE-T_streams_	PICo map
		ICE-T_ROI_i_	
		Waypoint region: ICE-T ROI_i-2_	
		(Any other waypoint, exclusion or termination masks)	
3	Threshold the streamlining result at ICE-T_threshold_	PICo map (from Stage 2)	New binary region ICE-T_ROI_i+1
		ICE-T_threshold_	
4	Check for continued growth of seed region:	ICE-T_ROI_i_	Growth flag
	Is (ICE-T_ROI_i+1_) > (ICE-T_ROI_i)?	ICE-T_ROI_i+1_	= 0 (no ROI growth)
			= 1 (ROI growth)
5	If Growth flag = 1, then i = i+1		ICE-T_ROI_i
	Goto Stage 2		
6	Display resultant segmented tract		ICE-T_ROI_I

ICE-T_threshold_: The level of “connection probability” (typically scaled between 0 and 1), above which a voxel must rise for it to be considered part of a significant connection, and therefore to be appended to the seed region from which the streamlines were initiated. Applied at each iteration of the feedback loop.

ICE-T_streams_: The number of streamlines emitted from each voxel in the seed region during the region-growing steps.

ICE-T_iterations_, I: The number of times that the seed region is iteratively grown.

The ICE-T framework consists of iteratively growing the seed region-of-interest (ROI), ICE-T ROI_i_, (where ‘i’ indicates the iteration count) along the tract branches it comes across, as outlined in Table 1. At each step, a PICo map [9] is generated and then thresholded at ICE-T_threshold_ to produce ICE-T ROI_i+1_. This ROI, if it has increased in size, is then fed back to the tracking step where it is used as the updated seed region for the next iteration. By employing the connection-confidence value of a voxel as the predicate, a connectivity constraint is automatically imposed upon ICE-T ROI_i_ and all its voxels are thereby classified as being highly connected to one another and hence also to the original seed region. In addition, the connectivity constraint is enhanced at each iteration by the application of a streamline waypoint region through which all streamlines are required to pass. The waypoint region for iteration ‘i’ is defined as ICE-T ROI_i-2_ (equivalently the seed region for iteration ‘i-1’). This guarantees that the original seed region will be included in the final segmented tract. For efficiency, the streamline computations are stored between iterations meaning that at each iteration only streamlines from the newly-included voxels need to be generated. The final grown seed region ICE-T ROI_I_ (where ‘I’ represents the total number of iterations performed), representing the segmented tract system from the original seed, can then be used for tract-based analysis. The resultant ICE-T ROI_I_ is not a PICo map, but instead each voxel's value represents an index of connection confidence with every other voxel within the tract, and is hereby defined as the Intra-Tract Confidence.

## Methods

### Ethics Statement

#### Animal data

All procedures followed “Guidelines for the Care and Use of Experimental Animals” and were approved by the Danish Animal Experiments Inspectorate.

#### Human data

The participant signed an informed consent following the guidelines of the declaration of Helsinki. The study protocol (KF 01 – 131/03) was approved by the local ethics committee (“De Videnskabsetiske Komiteer for Københavns og Frederiksberg Kommuner”).

### Data Acquisition and Pre-Processing

#### i) Ex-vivo pig brain

The data, including ground-truth seed regions defined by tracer injection, from three young and normal Göttingen mini pig brains (P1, P2 and P3), as used in [16], were re-used here to permit comparison against the validated results reported therein.

MRI data were acquired ex vivo on a 4.7T Varian MR scanner using a pulse gradient spin echo (PGSE) sequence with single line read-out using the following parameters: TR = 6500 ms, TE = 67.1 ms; matrix  = 128×128, in-plane resolution  = 0.51×0.51 mm^2^. Diffusion sensitisation gradient duration δ = 27 ms, time between gradient-pulse onsets Δ = 33.5 ms, gradient strength 56 mT/m. A slice thickness of 0.5 mm, gap 0.5 mm and two sets of 35 interleaved slices ensured whole brain coverage. NEX = 2. The pig brain datasets consisted of 3×b = 0 s/mm^2^ and one b-value of 4009 s/mm^2^ (chosen as specified in [21]), acquired in 61 non-collinear directions as available in Camino [22]. Before MR scanning the tissue was temperature stabilised to room temperature and a dummy run lasting 15 hours ensured that no short-term instabilities were introduced into the final diffusion MRI dataset [21].

The application of a spin-echo diffusion sequence, and the absence of both physiological and subject-generated motion, minimised the distortions in the ex vivo data. Visual inspection confirmed that no additional processing of the ex vivo data was required prior to tractography [21]. To limit subsequent analysis to brain tissue only, a brain mask was generated via summation of all diffusion images followed by application of a suitable threshold.

For tractography, we used the same hand-drawn seed regions as defined in [16] based upon the following tracer injection sites: right prefrontal cortex (PFC), right somatosensory cortex (SC) and the left motor cortex (MC). The datasets, including the seed regions, are freely available on (http://dig.drcmr.dk).

#### ii) In-vivo human brain

A single healthy volunteer (right-handed female, age 21 years), with no history of neurological or psychiatric disorders, or a family history thereof, nor hypertension, was recruited.

An in-vivo diffusion MRI dataset was acquired on a 3T Trio Siemens MR scanner with an eight-channel head coil (Invivo, FL, USA) using a twice-refocused diffusion-weighted sequence to minimise eddy current distortion [23] with the following parameters: TR = 8200 ms, TE = 100 ms, matrix  = 96×96; NEX = 1, GRAPPA acceleration factor  = 2.5, number of reference lines  = 48, isotropic 2.3 mm voxels and 61 slices (no gap) ensuring whole brain coverage. Ten b = 0 s/mm^2^ and one b-value of 1200 s/mm^2^ were acquired along the 61 non-collinear directions available from Camino [22]. Additionally, a gradient-echo-based field map sequence (TR = 530 ms, TE(1)  = 5.19 ms, TE(2)  = 7.65 ms, FOV = 256×256 mm, matrix 128×128, 47 slices with no gap, voxel size 2×2×3 mm, NEX = 1, acquisition time  = 2.18 min) was acquired to correct geometric distortions caused by B0 magnetic field inhomogeneities, and a 3-D whole brain T1-weighted magnetization prepared rapid acquisition gradient echo (MPRAGE) scan (1 mm^3^ isotropic voxels, FOV 256 mm, matrix  = 192×256×256, TR = 1540 ms, TE = 3.93 ms, TI = 800 ms, flip-angle  = 9°) was acquired for the generation of tissue segmentations and thereafter a brainmask.

Intra-volume subject motion and the undesired stretching or shearing caused by eddy-current build-up were simultaneously corrected for by estimating a 12 parameter affine model [24] to co-register the DWI images with the first b = 0 image of the sequence. Field inhomogeneity distortions, causing a geometric displacement of voxel intensities along the phase encode direction of the images, were addressed by acquisition of a field-map. The field-map correction [25] shipped as part of SPM8 (www.fil.ion.ucl.ac.uk/spm) was then used to estimate the voxel displacement map. The DWI images were then re-sliced into the space of the first b = 0 image via cubic b-spline interpolation within SPM8, with voxel intensities per volume scaled by the Jacobian determinant of the calculated transformation matrix. Finally, the rotational part of the affine model was applied to reorient the gradient directions [26].

A brain mask was generated in the diffusion image space using the grey- and white-matter (GM & WM) segmentations generated by SPM8 from the MPRAGE T1 scan.

For tractography, a cubic seed VOI was specified (by ML) using Matlab, defined so as to approximate the left subcortical motor area. The location and size of the VOI was defined following tractography seeded in the brainstem.

### Tractography

#### i) Probabilistic Tractography

For this work we chose, for comparative purposes, to use the same probabilistic tractography method as that was employed in Dyrby et al [16] i.e. the multi-tensor fibre reconstruction algorithm [3], [8] implemented in the Camino software package [22]. This comprised a voxel classification procedure [27] to allocate the likely number of component fibre directions within each voxel, followed by fitting of a multi-tensor model using a maximum of two fibre directions per voxel. This was then followed by streamline propagation from the centre of every voxel, using the FACT streamlining method [28] (with an inner-product threshold of 0.5, imposing a maximum within-voxel curvature of 60 degrees) to generate PICo maps [9]. The number of streamlines emitted from each voxel within the original seed region was 64,000 [16], whilst 25,000 streamlines were employed for the human in vivo data. These numbers are far greater than those often employed (5,000–10,000) and were chosen so as to ensure that resultant PICo values could not be attributed to poor sampling of the proximal tract network. However, due to PLD, poor sampling of the distal tract will occur even with this number of streamlines [29]. Streamlining was restricted to brain-only voxels via a brain-mask.

For comparison, results from tractography were subjected to a linear correction for PLD as described in [18]. Here the PICo values obtained from tracking along a given tract of interest are multiplied by their voxel distance from the seed region.

#### ii) Probabilistic Tractography With ICE-T

For the ICE-T Framework, we employ the same setup and parameters for probabilistic tractography as described above, with the exceptions that the number of streamlines was defined by the ICE-T_streams_ parameter, and with the addition of the ICE-T_threshold_ parameter, as described in Table 1.

The ICE-T Framework was implemented in Matlab (Mathworks Inc.) as a data-flow wrapper, handling file management and with calls to the required tractography functions from the Camino package [22].

An initial experiment was performed to empirically investigate the combined impact of both ICE-T parameters, ICE-T_threshold_ and ICE-T_streams_, using a single pig-brain dataset (P1) and sampling the following values:

ICE-T_threshold_: [0.001, 0.005, 0.01, 0.05, 0.10, 0.15, 0.20, 0.25, 0.30]

ICE-T_streams_: [1, 3, 5, 10, 20, 25, 50, 100, 250, 500].

To reduce processing time and storage demands when investigating the ICE-T parameter space, a file repository of 500 projection streamlines per voxel for dataset P1 was generated a priori, from which streamline samples could be randomly drawn as required. This experiment permitted the derivation of a single value for the ICE-T_streams_ parameter, one which could then be employed for all three pig-brain datasets.

Subsequently, the impact of the ICE-T_threshold_ parameter was investigated using this fixed value of ICE-T_streams_ on all three pig-brain datasets, with the following values:

ICE-T_threshold_: [0.005, 0.01, 0.015, 0.02, 0.025, 0.03, 0.035, 0.04, 0.045, 0.05, 0.06, 0.07, 0.08, 0.09, 0.1, 0.11, 0.12, 0.13, 0.14, 0.15, 0.16, 0.17, 0.18, 0.19, 0.2, 0.21, 0.22, 0.23, 0.24, 0.25]

### Analysis

To assess the degree of PLD along a selected tract, we select a line-of-interest (LOI) along its midline (see below). To delineate the LOI, we determined the most-likely pathway by selecting the canonical streamline, S_canonical_, from a collection propagated from the seed region to a waypoint. The canonical streamline, S_canonical_, was estimated as that which had the greatest number of points of agreement with all other streamlines. For this we used the first 100 successful streamlines. For analysis and subsequent comparison of connection-confidence values produced with and without ICE-T, S_canonical_ was then employed as the LOI to extract the tract midline data.

In the pig-brain datasets, S_canonical_ was derived from validated pathways described in [16], using the seed regions and waypoints specified therein. The LOI emanating from the SC region was chosen as an example due to its length and its passage through the complex region comprising the substantia nigra.

## Results

### ICE-T Parameter Selection

#### i) ICE-T_streams_ Parameter

**Figure 1:**
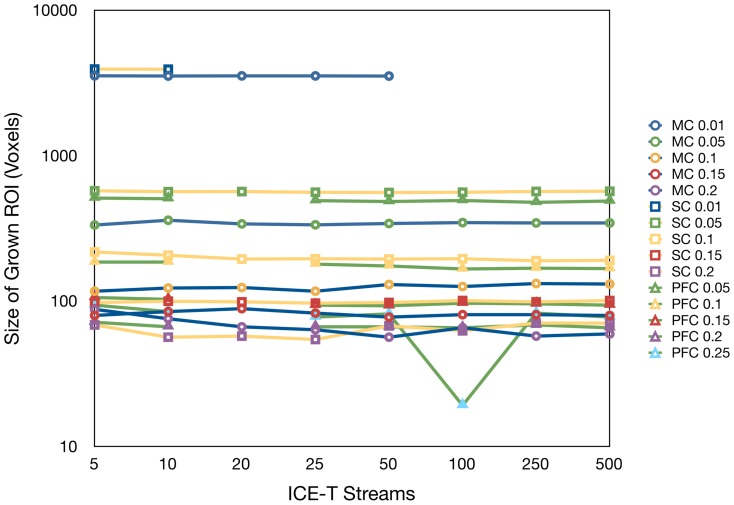
The impact of the ICE-T_streams_ parameter. Curves show the parameter's effect upon the size of ICE-T_ROI_I_ for various choices of the ICE-T_threshold_ parameter and seed regions (detailed in the legend). Seed regions are labelled as MC (motor cortex), SC (somatosensory cortex) or PFC (prefrontal cortex).

The first experiment investigated the combined impact of both ICE-T parameters. Figure 1 shows that the ICE-T_streams_ parameter has no global effect upon the size of ICE-T ROI_I_, however the latter does decrease with increasing ICE-T_threshold_. A slight variation in the size of the ICE-T ROI_I_ can be observed in the PFC and MC between 5 and 10 streamlines. An outlier is noted at a single point for the PFC at ICE-T_streams_ 100 and ICE-T_threshold_ 0.25. For the remaining experiments, we fixed the ICE-T_streams_ at 20 streamlines, as a compromise between having a sufficient number of streamlines whilst minimising computational resources.

#### ii) ICE-T_threshold_ Parameter

**Figure 2:**
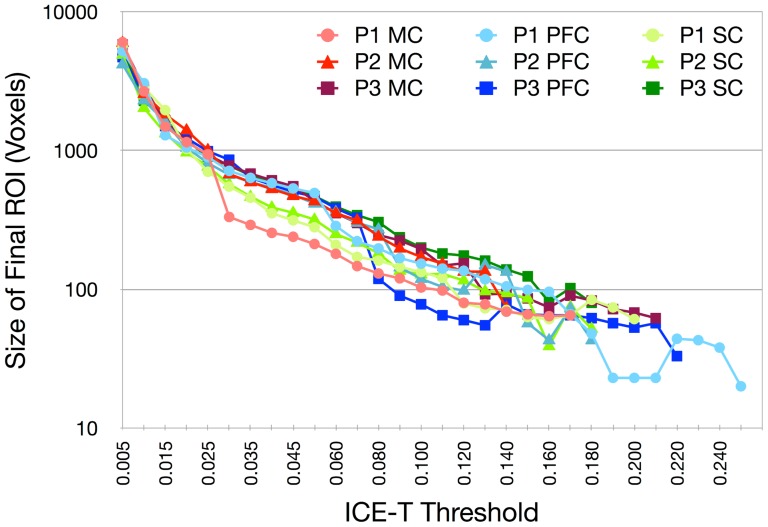
The impact of the ICE-T_threshold_ parameter. Curves show the parameter's effect upon the size of ICE-T_ROI_I_ with the ICE-T_streams_ parameter fixed at 20 streamlines, shown for each of the three ROIs (MC, PFC and SC) in each of the three ex-vivo datasets (P1, P2 and P3).

**Figure 3:**
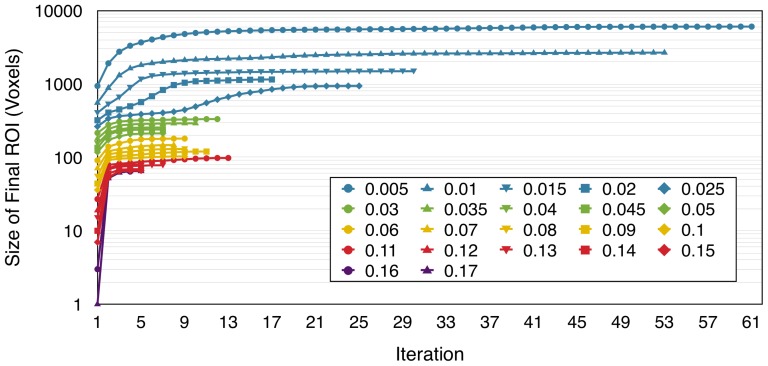
The impact of the number of ICE-T iterations performed. Curves show the effect of iteration number upon the size of ICE-T_ROI_I_ for dataset P1, using the MC seed region, and for various values of the ICE-T_threshold_ parameter. For clarity, a log scale has been employed for the vertical axis.

For any seed region, the ICE-T_threshold_ parameter significantly impacts the size of the ICE-T ROI_I_ as shown in Figure 2. The size of ICE-T ROI_I_ increases linearly as the ICE-T_threshold_ is reduced, down to around ICE-T_threshold_ = 0.025. Below this threshold, the increase is greater and non-linear, most likely due to the incorporation of adjacent tract networks into the segmented network along with a consequential increase in the number of false positives. For very low ICE-T_threshold_ (approx. <0.001), ICE-T ROI_i_ always grows at each iteration, and hence never generates a specific segmented tract network (results not shown). However, as shown in Figure 3 for the MC seed, selecting an ICE-T_threshold_ above 0.005 results in a stable ICE-T ROI_I_. Figure 3 also shows the general decrease in the number of iterations required to reach stability as ICE-T_threshold_ is increased. Further iterations provide no additional change, and the ICE-T halts at this point. The resultant ICE-T ROI_I_ therefore represents a segmented tract system emanating from the seed. Similar results are obtained for SC and PFC (results not shown). In the following experiments the number of ICE-T iterations is automatically limited, determined by the point at which the ICE-T ROI_i_ and ROI_i-1_ show no size differences, and hence the number of iterations is not a parameter in itself.

**Figure 4:**
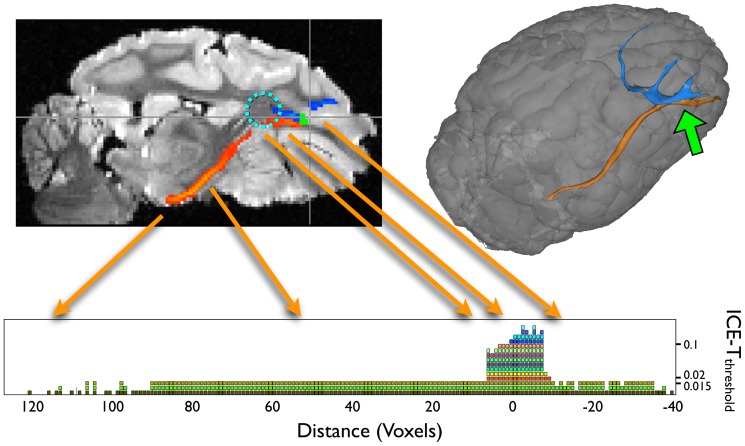
Illustration of the ability of ICE-T to penetrate through a complex region. The figure shows how ICE-T successfully propagates through a known crossing-fibre region (centrum semi-ovale, light blue dotted circle, upper left panel) when seeded from the SC region (green region, upper left panel) of dataset P1. The dark blue region (upper left panel) shows the results using ICE-T_threshold_ of 0.02, and the red region (upper left panel) for ICE-T_threshold_ of 0.015. The graph (lower panel) shows the spatial extent of the ICE-T_ROI_I_, sampled along the canonical streamline, from the seed region (defined as Distance  = 0) as a function of both distance from the seed region, and of the value of the ICE-T_threshold_ parameter. Here a coloured voxel represents that the segmented ROI was present at the given threshold and distance from the seed. Each threshold level is coloured differently for clarity. Once the ICE-T_threshold_ parameter falls to 0.015 and below (lower three rows), the region-growing penetrates past the complexity and continues on to extract the distal portion of the tract. The 3D rendering (upper right panel) shows the ICE-T results at the same two thresholds (0.02 in blue and 0.015 in orange). The seed region is located at the site of the green arrow (upper right panel).

The spatial growth of the ICE-T ROI_I_ from the SC seed region along the canonical streamline of the corticonigral tract that projects through a complex crossing fibre region (centrum semiovale) is illustrated in Figure 4. With this seed region, selection of ICE-T_threshold_>0.015 causes the region growing to halt at this complex region. However, ICE-T_threshold_ values below this level permit the ICE-T ROI_I_ to grow along the entire canonical streamline. Similar ICE-T_threshold_ values were also found for this seed region in the P2 & P3 datasets (results not shown).

### Tract Segmentation

**Figure 5:**
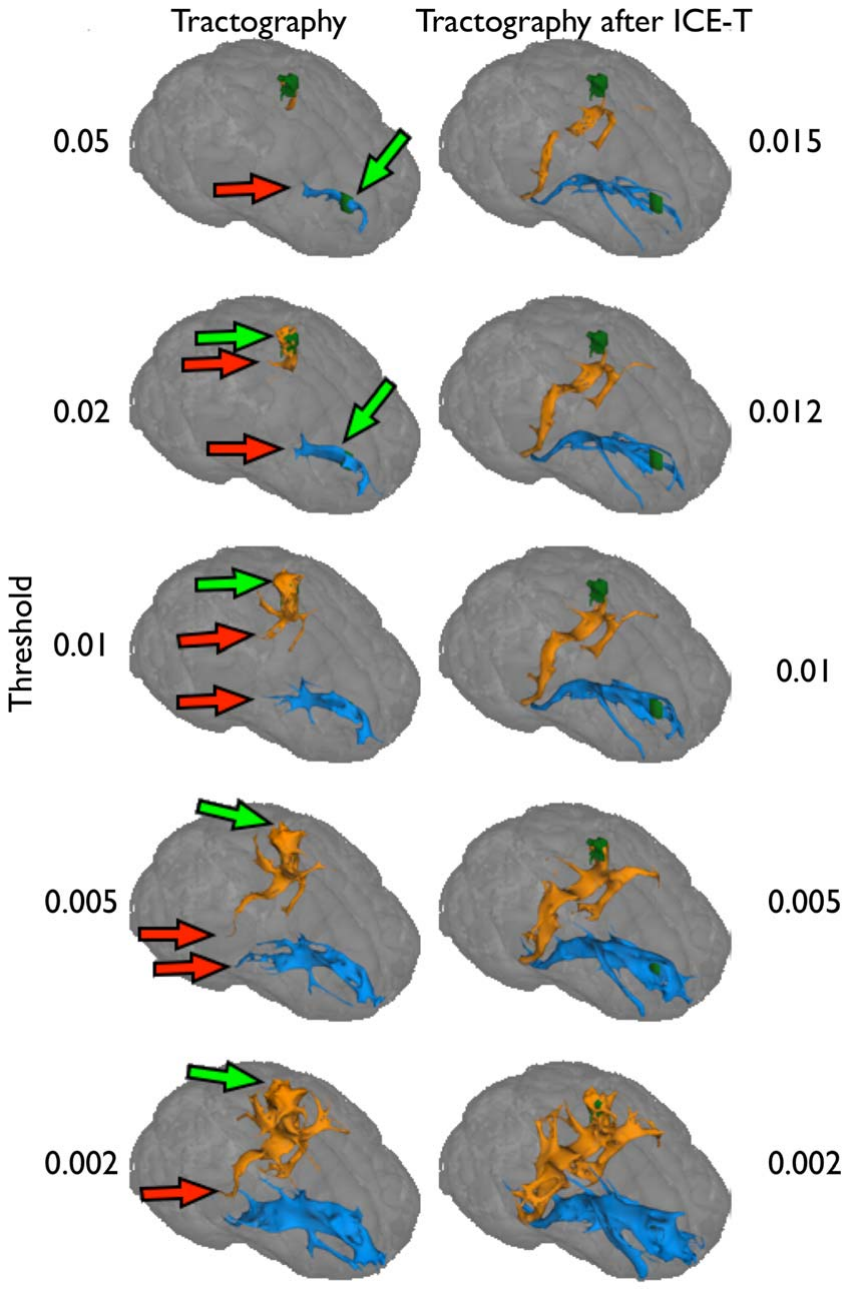
Comparison of tractography with and without ICE-T. Tractography is seeded from both the MC & PFC seeds (shown in green) of dataset P1.(Left Panel) Tractography without ICE-T (i.e. directly with the seed ROI) using N = 64000 streamlines per voxel and then visualised using the following thresholds (from top)  = 0.050, 0.020, 0.010, 0.005, 0.002.(Right Panel) Tractography with ICE-T ROI_I_ (generated using ICE-T_streams_ = 20, ICE-T_threshold_ = 0.01) used as seed, and then visualised at the following thresholds (from top)  = 0.015, 0.012, 0.010, 0.005, 0.002.Green arrows indicate areas demonstrating “near-seed flare”. Red arrows indicate premature termination of the tract ROI due to the PLD effect causing the PICo values to fall below the applied threshold.

Figure 5 shows tractography performed using only the seed ROI versus using the ICE-T_ROI_I_, thresholded at different levels. In order to extract the entire tract without using ICE-T i.e. using seed ROI (left column), thresholding of the obtained results would require application of a very low global threshold (<0.010). Using the seed ROI, the effect of low thresholding produces a near-seed flare, reflecting the high proportion of false positive connections found close to the seed, where the sampling is still sufficient (green arrows, Figure 5). Distally, due to the PLD effect, the false negatives become dominant, as evidenced by the sudden termination in white matter of those tracts that survive the thresholding (red arrows, Figure 5). In contrast, the results after application of ICE-T do not show such behaviour, and instead generate a segmentation of the tract network at each threshold. Notably, because a grown ICE-T ROI_I_ is used for tracking, then lowering the subsequent global threshold just broadens the cross-sectional area of the tract system. Near-seed flare effects are not present, and no sudden termination of tracts in white-matter are seen, indicating a reduction in the PLD effect.

**Figure 6:**
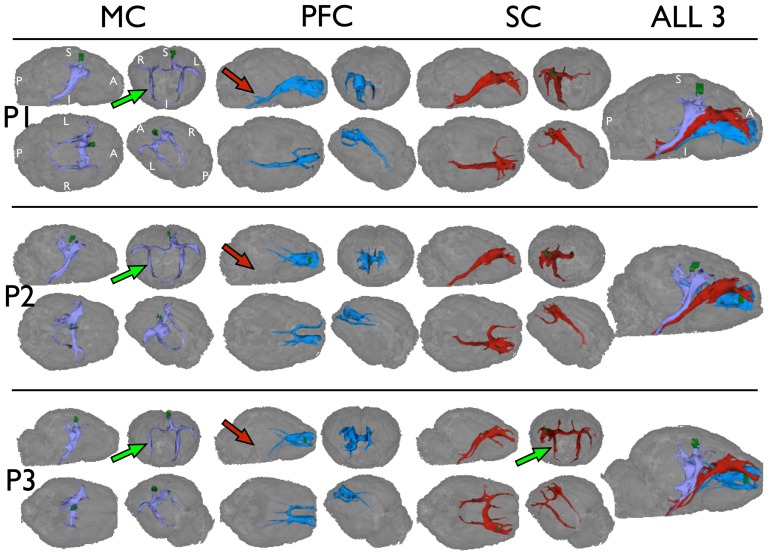
Tractography with ICE-T from each of the three ROIs (MC, PFC and SC), for each pig brain (P1, P2, P3). Parameters: ICE-T_streams_ = 20, ICE-T_threshold_ = 0.005, results rendered at 0.005. Data show the glass brain of the unweighted diffusion image as anatomical reference.

This demonstrates the potential for a common ICE-T_threshold_ which can be used across different subjects and seed regions, as illustrated in Figure 6 for an ICE-T_threshold_ of 0.005. Here an extensive tract network is segmented for all 3 seed regions and shows similar, though not identical, structures across all datasets (P1, P2, P3). Exceptions can be seen in, for example, the projections from the PFC seeds towards substantia nigra in P1 vs those from P2 and P3 (Figure 6, red arrows). Importantly, however, the cross-sectional sizes of the longer tracts appear consistent along their length, inferring an independence of the results to path length.

However, deviations from expected results do occur around complex regions. For example from the MC and SC seeds, false positive contralateral projections towards the internal capsule are observed, and when this is the case, they mirror completely the ipsilateral tract network (green arrows, Figure 6). As could be expected, the incorporation of just a portion of any false positive branch leads its extraction up to a cortical region.

### Tractography

The following compares the tractography results from the original seed regions (SC, PFC and MC) with those derived from a seed defined by the segmented tracts provided by ICE-T with an ICE-T_threshold_ of 0.005.

**Figure 7:**
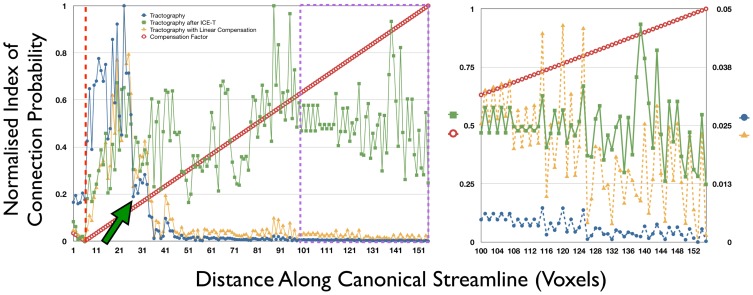
Comparison of along-tract profiles after tractography, with and without ICE-T, versus linear compensation. Curves show the variation of PICo values (scaled to [0,1]) along the canonical streamline from the MC seed region (red dashed vertical line) on dataset P2 for tractography with ICE-T (green), without ICE-T (blue), and tractography with linear compensation (orange). Right panel shows a zoomed portion of the main graph, delineated by the purple dashed border, where both the tractography without ICE-T (blue) and tractography with linear compensation (orange) are now drawn according to the scale on the right axis. Dataset details: ICE-T performed using ICE-T_streams_ = 20, ICE-T_threshold_ = 0.005, number of ICE-T iterations until stability  = 41. Tractography without ICE-T was generated using 5000 streams per voxel. A drastic fall-off in PICo values can be observed (green arrow) for tractography results without ICE-T soon after the streamlines exit the seed region due to their encountering a complex region. No such effect is seen for the results with ICE-T. For a tract reference, see the 3D render of this tract in Figure 6, P2, MC seed region.

The tractography PICo values in Figure 7 show a substantial drop in the area of the centrum semiovale, located at tract distance of 40 voxels from the seed, and subsequently show a continuing decrease towards the projection site, indicating the presence of PLD. It is also apparent how the combined effect of PLD and anatomically-related obstacles impose a non-linear behaviour upon the PICo values as a function of distance from the seed. Although the linear compensation factor is initially able to correct for distance after the seed region (approximate distance: 25–35 voxels), it is unable to compensate for the non-linear PLD effect introduced in the centrum semiovale and the values on the distal side remain low. In contrast, although the ICE-T results show variations along the length of the canonical streamline, there is no overall drop in Intra-Tract Confidence values related to such underlying complex regions.

### In-vivo human brain

**Figure 8:**
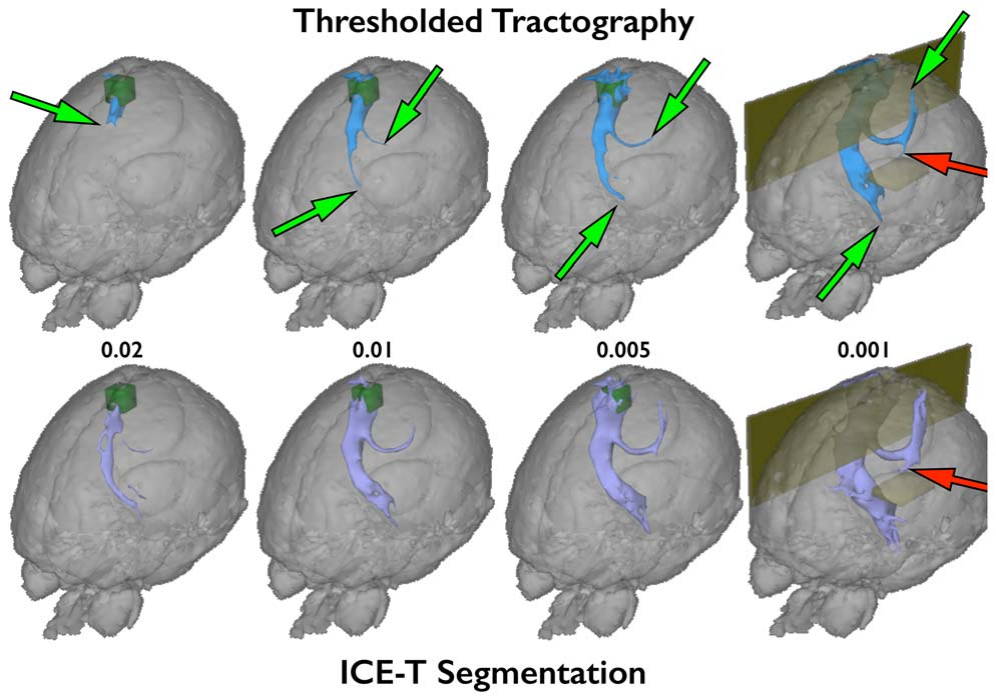
Comparison of thresholding tractography results, obtained with and without ICE-T, in a human in vivo subject. Both results are generated from a cubic seed (dark green) placed approximately in the left MC region. Tractography without ICE-T used the original cubic seed ROI as the seed (25,000 streamlines, blue, top row). Tractography with ICE-T used the ICE-T ROI_I_ as seed (ICE-T_threshold_ 0.01, ICE-T_streams_ 20, purple, bottom row), shown here at various rendering thresholds (0.02, 0.01, 0.005, 0.001).The path-length dependency is very pronounced in the tractography results without ICE-T (top row), evidenced by the movement of the end-of-tract point (green arrows) as a function of the applied threshold. Probable false-positives are seen in tractography both with and without ICE-T around the descending portion of the contralateral CST (red arrows). These can be addressed in the conventional manner by the introduction of exclusion masks (dark green box and plane) that terminate and remove any streamlines that propagate through them. Here two are shown for both methods (last column) - one along the mid-sagittal plane and one in the contralateral CST. The former is to prevent streamlines crossing between the hemispheres at the cortical level dorsal to the corpus callosum due to the high partial volume effect. The latter is to prevent segmentation of a known false-positive branch of the contralateral CST.

Results from the application of ICE-T to the in vivo human dataset are shown in Figure 8, using parameters within the same range as those determined to be applicable to the ex vivo dataset (ICE-T_threshold_ = 0.01 and ICE-T_streams_ = 20). Tractography without ICE-T employed the seed ROI, whereas that with ICE-T used the ICE-T ROI_I_ as seed. The ICE-T tracts show, as in the ex vivo data, a uniform cross-sectional size along their length as a function of threshold. In contrast, such features are not seen in the tracts generated from probabilistic tractography without ICE-T due to the effects of PLD, as was also observed in the ex vivo pig brain. A possible false positive can be observed in the ICE-T results, seen as a descending tract in the region of the contra lateral capsula interna (red arrow, Figure 8). Just as in tractography, we can explicitly remove false positives by using exclusive ROIs. After placing an exclusive ROI (small dark yellow region in Figure 8) contra-laterally, superior to the capsula interna region, the false positive projection can be removed.

**Figure 9:**
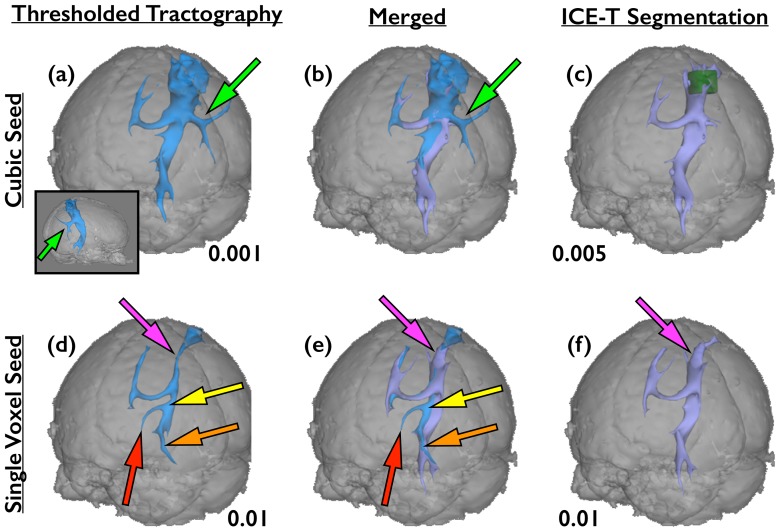
Tractography in a human in-vivo dataset, with and without ICE-T, showing dependency upon the size of the seed ROI. Top row ((a), (b), (c)) shows the same results as for Figure 8, but from a posterior viewpoint. From this angle it is also clear how the tractography without ICE-T using the cubic seed also generates a lateral cortical branch ((a), (b): green arrow). Inset on (a) shows lateral view from the right side, highlighting the posteriorly-directed angle of the branch.Bottom row ((d), (e), (f)): tractography results from a single voxel seed within the left MC, using the same parameters as for the cubic seed. As for the cubic seed, the rendering thresholds have been selected so as to generate comparable propagation of the tractography into the contralateral ascending portion of the CST. In the tractography results without ICE-T ((a), (d)), the ipsilateral descending portion follows a more medial route than the results using ICE-T ((c), (f)), as can be seen on the merged views ((b), (e)). Further inspection of these results indicates that the streamlines diverge from the CST around the level of the ventricles and seem to instead pick-up a periventricular route through the medial thalamic nuclei ((d), (e): yellow arrows). The streams then diverge, following a descending route close to the CST ((d), (e): orange arrows), and a medial route along the anterior thalamic radiation ((d), (e): red arrows). The ICE-T results correctly follow the CST from both seed areas.

Figure 9 illustrates the impact of the seed region specificity and size on the tractography results for the human in vivo data. The global thresholds for these results were chosen so as to match the segmented tracts for their distal propagation into the contralateral ascending portion of the cortical spinal tract (CST). The top row shows the results using the same cubic seed region as used in the Figure 8 – a cubic area centred approximately over the left motor cortex. The lower row shows the results using only a single voxel seed, chosen from within the cubic seed. Whilst the ICE-T results show minor impact of the choice of seed used, those of the probabilistic tractography without ICE-T demonstrate several differences (Figure 9). Firstly, with the cubic seed, the degree of near-seed flare is substantially greater than that after ICE-T (Figure 9(a) vs. 9(c). Note that the green seed region is enveloped by the tract in 9(a) but not in 9(c)). Secondly, the PLD effect is greater when using the single-voxel seed (Figure 9(a) vs. 9(d)). Thirdly, the segmented tracts differ slightly in both shape and extent for the two seed regions (Figure 9b). Using the cubic seed produced a lateral cortical branch not seen for the single-voxel seed (Figure 9(a)(b) vs. 9(d)(e), green arrows). In contrast, the single-voxel seed produced an ipsilateral medial branch (Figure 9(d)(e), red arrows) that appears to divert from the CST around the level of the corpus callosum (Figure 9(d)(e), yellow arrows) and instead follow the anterior thalamic radiation. Inferiorly, the descending portion (Figure 9(d)(e), orange arrows) also appears to follow a different route than the CST, before terminating prematurely.

## Discussion

We have shown that PLD can arise as a non-linear effect modulated by tissue complexity, and that some of the effects imposed by PLD upon probabilistic tractography are the near-seed flare (false positives), and reduced distal propagation (false negatives), confounds which have been speculated to bias structural connectivity analysis ([13], [30]). We have introduced the ICE-T Framework as a generalizable wrapper around existing methods and demonstrated its ability to mollify the universal PLD confounds in segmented tracts. The results presented herein suggest that using methods such as ICE-T that address the PLD confound will benefit the statistical robustness for a wide range of group statistics, such as structural connectivity analysis and advanced tract shape models

### The PLD confound

At present, the PLD confound is rarely addressed. Indeed, it is common for the threshold-level of probabilistic streamline tractography experiments to either be left extremely low (giving ‘near-seed flare’ effects, as observed in Figure 5), or to select a threshold which perceptually segments-out the tracts of interest. Both approaches are subjective, and therefore obviate meta- or group-analysis. As noted by [19], PLD imposed problems for the delineation of the tracts of interest in their group study, and so to compensate, they found it necessary to employ several extra heuristic constraints.

Linear propagation distance factors have previously been used in an attempt to correct for the PLD effect [18], [17]. However, we observed how anatomical complexities, e.g. centrum semiovale, which cause barriers to streamline propagation, compounded the PLD effect in a non-linear manner (Figure 7). We demonstrate how linear compensation techniques cannot correct for the non-linearity of the PLD effect, in contrast to ICE-T. The ICE-T approach has the additional advantage of being independent of the image resolution, unlike the PLD effect, which will increase with the number of propagation steps (and thereby voxels), required to reach the target. As such, the impact of PLD is likely to increase as future studies permit the use of higher resolution imaging techniques.

### Benefits of the Iterative Process

In conventional tractography, the PLD imposes a limit upon how far a streamline is likely to propagate away from a seed. As a consequence, increasing the number of streamlines used cannot improve the propagation performance [29]. In contrast, application of ICE-T generates a delineation of the tract, thereby iteratively distributing the seed voxels along its length. This has the consequence that average streamline path-lengths will be diminished, thereby causing the distribution of false positives and negatives along the length of the delineated tract to be more uniform. Naturally, the decrease in distal false negatives also means a concurrent increase in distal false positives. Note that the presence of distal false positives actually reflects the removal of PLD, because such errors are most likely to occur where the sampling of the uODFs is sufficient.

The tracking algorithm reported in [31], based upon Time-Of-Arrival (TOA) maps, has some similarities to the iterative nature of ICE-T, but was developed as a way to improve tracking performance of streamlines that met with problematic regions such as those containing crossing fibres. The procedure includes an iterative region-growing step that is similar to the approach presented herein, and although they did not report any such findings, it too may show an improvement to the PLD effect. However the approach cannot be generalized to existing tractography methods.

Prior knowledge in the form of waypoints and exclusion ROIs can be used with ICE-T to reduce the false positives as in conventional tractography. Similarly, prior knowledge can be applied to constrain the segmentation to specific fibre bundles emanating from a seed. Furthermore, as shown in Figures 5 & 8, the tracts generated by ICE-T have more uniform cross-sectional areas along their entire length, and the extent of the cross-section can be controlled by the global threshold parameter. Hence the ICE-T tract volumes are suited for use as binary masks to generate sample volumes (VOIs) for tract-oriented statistics, e.g. [32].

### Generalisability

It must be highlighted that probabilistic tractography with ICE-T is not a new tracking algorithm *per se*, but a generic framework applicable to most probabilistic streamlining methods. As such it is able to benefit from their long-standing methodological developments, and their individual advantages and disadvantages.

ICE-T has a further benefit of generalizing the parameter choice. Conventional tractography, besides the definition of a seed region, requires the specification of the number of streamlines and usually the global threshold, applied to the probabilistic results in order to delineate the desired tracts. Aside from the seed region, the parameters of the ICE-T Framework are ICE-T_threshold_ and ICE-T_streams_, used to generate ICE-T_ROI_I_, along with the subsequently-applied global threshold. The streamline parameter has a different purpose in the two methods. In conventional tractography, the number of streamlines is chosen heuristically in an attempt to sufficiently sample the entire tract. Liptrot & Dyrby [29] demonstrated how increasing the number of streamlines (typically up to 5000) simply increased the voxelwise connectivity probabilities, and so was unable to address the PLD. However when using ICE-T, we have shown how far fewer streamlines are required (approximately 20) - any more than this show minor additional benefits and simply add to computational burden. This is because at each iteration only the local tract environment needs to be sufficiently sampled. The ICE-T_threshold_ parameter controls the minimal degree of connectivity confidence required that new voxels must attain to be incorporated into the growing seed. We have shown how choosing ICE-T_threshold_ at approximately 0.01 permits segmentation of the tract network. Selection of a too high ICE-T_threshold_ hinders the growth of the seed region through complex regions, e.g. centrum semiovale. In contrast, too low a value of ICE-T_threshold_ will lead to growth of the seed region outside of the relevant tract network. The exact choice of ICE-T_threshold_ will depend upon several factors, including acquisition parameters (e.g. imaging sequence, resolution), but especially the tractography method as well as the topology of the particular tract network being analysed.

The tracking results obtained with ICE-T show wide agreement with those obtained in [16] using in vivo tracers. However omissions were also noted, for example the absence of the corticonigral projections from the PFC region for datasets P2 and P3 (Figure 6, red arrows). Although previous work [16] has successfully delineated these tracts for this dataset using both in-vivo tracers and tractography, the latter was achieved via the application of waypoint constraints. This suggests that local complexities may have prematurely halted the tractography using ICE-T, and that a reduction of the ICE-T_threshold_ may be needed to permit successful penetration into the distal portion of the tracts. This underlines that external factors such as tractography method and dataset parameters (e.g. resolution, b-value) influence the selection of ICE-T threshold. However, we have shown how the parameters are generally transferrable to similar, ex vivo datasets (P1, P2, P3), and have also successfully applied it to an in vivo clinical dataset. The ICE-T parameters are not expected to be generalizable across tractography methods or acquisition parameters, however it is expected that they will also exhibit a stable range. In future work we will investigate the effect of various tractography methods upon the ICE-T parameters.

A major difference between tractography with and without ICE-T is that while the latter outputs a PICo map based upon tracking from a given seed region, ICE-T generates an Intra-Tract Confidence map of all connections within the segmented tract. Interpretation of the Intra-Tract Confidence map is therefore different from that of a PICo map. The direct interpretation of the values has not been considered herein. However, since a PICo map is a metric of streamline propagation from a seed region, it is affected by tract integrity, but is not a direct measure of it. In contrast, the Intra-Tract Confidence map from ICE-T is a metric that reflects the sum of connections from every member voxel, most of which will, by construction, lie within the ICE-T_ROI, i.e the segmented tract. However, this in turn means that it cannot be used directly for network analysis as it does not reflect a probability of being connected to the seed, but instead it can be used as a binarized version of the tract system emanating from the seed. The latter is often used for creation of structural connectivity matrices.

### Considerations

In tractography, streamlines are propagated in both directions from the seed region. It should be noted, however, that the ICE-T method we have implemented here is based upon the Camino toolbox and does not include directionality constraints applied to each ICE-T_ROI_i_ region. This infers the possibility that the segmented tract network might reflect bi-directional pathways along the entire delineated tract. If such behaviour is undesirable then a simple forwards-only directionality constraint could be applied at the end of each ICE-T iteration.

When specifying the initial seed region, we expect that any subset of voxels within the region of interest could be employed. Due to the region-growing feature of the seed region when using ICE-T, it is expected that the iterative region-growing will expand the seed to approximate the entire tract network. This same argument would also imply that care must be taken to ensure that over-inclusive regions are not employed as seeds. For example, we found false-positive lateral branching only in the results using the overly-large ROI when ICE-T is not applied (Figure 9a,b). This suggests that the imprecise delineation of an ROI (too large) could be a source of false positives in tractography, whereas ICE-T appears to be more robust to the precision of the ROI. In addition, as was clearly demonstrated in Figure 9f, a major advantage of the ICE-T method is the ability to segment a tract network using only a subset, even a single voxel, of the seed region of interest. Uniquely, using ICE-T with such a subset does not result in a penalty of increased PLD, and it is still able to segment out the same network as a much larger seed. This has obvious benefits for future clinical studies where accurate delineation of anatomical areas of interest to act as seed regions could be obviated and replaced by a selection of a single voxel within the known region. Such an approach is likely to be simpler and more reproducible as the margin for error will be a function of the region's size, and the selection could occur in those subregions where the confidence of correct localisation is highest.

## Conclusions

The impact of PLD on the results of probabilistic streamline tractography is a confound which should be considered. We have shown the non-linear spatial variation of PLD along any given pathway, challenging the application of a global threshold and introducing both false positives (near-seed flare) and false negatives (premature tract termination). We have shown how a novel re-appraisal of the probabilistic streamline tractography pipeline, termed ICE-T Framework (ICE-T), offers the possibility to segment tract systems without the problems imposed by PLD. With ICE-T, PLD issues are substantially reduced to the point where tract networks can be delineated using a global threshold, leading to a reduction in the PLD-related confounds. Importantly, ICE-T only addresses the PLD issue, and preserves all the characteristics of the individual tractography methods. It is recommended that future work should consider handling PLD in order to minimise the risk of bias in tract statistics and structural network analysis.
